# Molecular Characterization of Multidrug-Resistant *Yersinia enterocolitica* From Foodborne Outbreaks in Sweden

**DOI:** 10.3389/fmicb.2021.664665

**Published:** 2021-05-13

**Authors:** Philip A. Karlsson, Eva Tano, Cecilia Jernberg, Rachel A. Hickman, Lionel Guy, Josef D. Järhult, Helen Wang

**Affiliations:** ^1^Department of Medical Biochemistry and Microbiology, Biomedical Center, Uppsala University, Uppsala, Sweden; ^2^Department of Medical Sciences, Uppsala University Hospital, Uppsala, Sweden; ^3^Public Health Agency of Sweden, Solna, Sweden; ^4^Science for Life Laboratories, Uppsala University, Uppsala, Sweden; ^5^Department of Medical Sciences, Zoonosis Science Center, Uppsala University, Uppsala, Sweden

**Keywords:** *Yersinia enterocolitica*, WGS, AMR, Tn*2670*, *tet*B

## Abstract

The foodborne pathogen *Yersinia enterocolitica* causes gastrointestinal infections worldwide. In the spring of 2019, the Swedish Public Health Agency and Statens Serum Institut in Denmark independently identified an outbreak caused by *Yersinia enterocolitica* 4/O:3 that after sequence comparison turned out to be a cross-border outbreak. A trace-back investigation suggested shipments of fresh prewashed spinach from Italy as a common source for the outbreak. Here, we determined the genome sequences of five *Y. enterocolitica* clinical isolates during the Swedish outbreak using a combination of Illumina HiSeq short-read and Nanopore Technologies’ MinION long-read whole-genome sequencing. WGS results showed that all clinical strains have a fully assembled chromosome of approximately 4.6 Mbp in size and a 72-kbp virulence plasmid; one of the strains was carrying an additional 5.7-kbp plasmid, pYE-tet. All strains showed a high pathogen probability score (87.5%) with associated genes for virulence, all of which are closely related to an earlier clinical strain Y11 from Germany. In addition, we identified a chromosomally encoded multidrug-resistance cassette carrying resistance genes against chloramphenicol (*catA1*), streptomycin (*aadA1*), sulfonamides (*sul1*), and a mercury resistance module. This chromosomally encoded Tn*2670* transposon has previously been reported associated with IncFII plasmids in *Enterobacteriaceae*: a *Shigella flexneri* clinical isolate from Japan in 1950s, a *Klebsiella pneumoniae* outbreak from Australia in 1997, and *Salmonella enterica* serovar Typhimurium. Interestingly, we identified an additional 5.7-kbp plasmid with *tet*B (encoding an ABC transporter), *Rep*, and its own ORI and ORIt sites, sharing high homology with small *tet*B-*Rep* plasmids from *Pasteurellaceae*. This is the first time that Tn*2670* and *Pasteurellaceae* plasmids have been reported in *Y. enterocolitica*. Taken together, our study showed that the Swedish *Y. enterocolitica* outbreak strains acquired multi-antibiotic and metal-resistance genes through horizontal gene transfer, suggesting a potential reservoir of intraspecies dissemination of multidrug-resistance genes among foodborne pathogens. This study also highlights the concern of food-chain contamination of prewashed vegetables as a perpetual hazard against public health.

## Introduction

*Yersinia enterocolitica* is a foodborne zoonotic bacterium of global importance, able to cause severe gastrointestinal infections among people of all ages (Scallan et al., 2011; Batz et al., 2012; Bancerz-Kisiel and Szweda, 2015). *Yersinia* is grouped under *Yersiniaceae* where *Yersinia pestis*, *Yersinia pseudotuberculosis*, and *Y. enterocolitica* are recognized as important human pathogens (Smego et al., 1999; Adeolu et al., 2016). *Y*. *enterocolitica* is divided into six biotypes (1A/B-5) depending on physio- and biochemical properties and additionally into approximately 50 serotypes depending on antigenic variation of the lipopolysaccharides (Jagielski et al., 2002).

While yersiniosis historically has been associated with consumption of undercooked pork products or un-pasteurized milk, the growing trend of ready-to-eat (RTE) vegetables in industrialized countries has been linked to sporadic outbreaks of *Y. enterocolitica* (Lee et al., 2004; Sakai et al., 2005; Much et al., 2009; Macdonald et al., 2011; Rahman et al., 2011). As discussed during the amplified implementation of the Hazard Analysis and Critical Control Point (HACCP) system in the 1990s, in the raw food industry, RTE products were highlighted as products without any pathogen elimination step (Motarjemi and Käferstein, 1999). Even without pathogen elimination, HACCP remains a good resource to reduce risks in food production, but intensification and mass distribution have nonetheless permitted quick nationwide spread of introduced contaminants. International trade has correspondingly introduced longer food-supply chains and countless wholesalers, increasing the risk of breaking the cold chain with consequential growth of foodborne pathogens (Motarjemi and Käferstein, 1999; Söderqvist et al., 2017a, b). Wholesalers in the European Union are required to regularly sample RTE vegetables for *Escherichia*, *Listeria*, and *Salmonella*, but *Yersinia* remains continuously absent from routine testing (EU commission 2073/2005) and its AMR surveillance occurs only through rare *ad hoc* reports (Fàbrega et al., 2015).

In the spring (February–April) of 2019, the Swedish Public Health Agency (PHAS) and Statens Serum Institut (SSI) in Denmark independently identified an outbreak with the same genotypic cluster of *Y. enterocolitica* 4/O:3. Whole-genome sequencing (WGS) comparisons were made using the respective outbreak sequences, resulting in a cross-border outbreak being declared. The outbreak reached 57 positive cases, mainly in a younger age group (15–39) (National Veterinary Institute (SVA), 2019). A Danish case–control study recognized fresh spinach as the cause behind the outbreak, and a traceback of common producers identified an Italian supplier behind shared market batches (Espenhain et al., 2019). Following the first outbreak, a second outbreak (April–May) occurred with an additional 30 cases. *Y. enterocolitica* in the second outbreak was of identical bioserotype and sequence type (ST) type but clustered separately on single nucleotide polymorphism (SNP) analysis. No food-related origin could be established for the latter outbreak (National Veterinary Institute (SVA), 2019).

Whole-genome sequencing is now routinely used in outbreak investigations to identify ST types, clusters, pathogenomics, and resistance genes, but typically this high-throughput resource is limited to short-read data (Lynch et al., 2016; Nutman and Marchaim, 2019; Adzitey et al., 2020). Short-read libraries are precise and cost-efficient but do rarely provide long enough contigs for molecular epidemiological identification of moveable genetic elements and small plasmids (Koren and Phillippy, 2015). Long-read sequencing technologies can now be used in combination with short-read data to provide high-quality full-genome assemblies (Jain et al., 2016; George et al., 2017; Lemon et al., 2017). To provide insight in resistance and adaptive traits in *Y. enterocolitica* causing foodborne outbreaks, we combined Illumina HiSeq and Oxford Nanopore technologies’ MinION to generate and close whole genomes for five clinical strains, isolated in Sweden during the time of the two consecutive outbreaks in 2019. This is the first report of multidrug-resistant *Y. enterocolitica* identified from imported fresh spinach, raising concerns on food-chain contamination.

## Materials and Methods

### Bacterial Isolation and Identification

A total of five clinical isolates from the spring of 2019 were characterized. PHAS received isolates from the Swedish clinical microbiological laboratories for epidemiological typing, and three of these were used in the study (Espenhain et al., 2019). Y30 was derived from the spinach-related outbreak (March), Y108 was isolated from the second outbreak (April), and Y72 was collected during the outbreak periods (April) but did not cluster with any of the outbreak strains (SNP analysis, PHAS). Two additional uncharacterized isolates were collected from the Clinical Microbiology Lab at Uppsala University Hospital from the same period, Y_Mar (March) and Y_May (May). All five isolates are in this study referred to as outbreak strains. One well-characterized strain Y11 (chromosome: FR729477, plasmid: FR745874) was received from the Leibniz Institute (DSM 13030) for comparative use. The Swedish strains were delivered in transport swabs with amies medium charcoal (SARSTEDT), streaked onto Cefsulodin Irgasan Novobiocin (CIN) agar with *Yersinia* supplement and incubated for 24 h at 26°C. A one-μl streak of confirmed *Yersinia* colonies was cultivated in Trypticase Soy Broth (TSB) for 24 h in shaking incubators at 26°C. Aliquots were saved in TSB-DMSO (10%) and stored in −80°C freezers until further use. Ethical approval was not required as the investigation was performed under a mandate of PHAS in its remit to undertake outbreak investigations regarding national communicable disease control in the interest of public health.

### Whole-Genome Sequencing

Stocks were cultured on Trypticase Soy Agar (TSA) and in one ml TSB at 26°C overnight. Genomic DNA was extracted from overnight cultures with the MasterPure DNA purification kit (Epicentre, Lucigen) according to the manufacturer’s instructions. Purified DNA was analyzed and quantified with 2100 NanoDrop Spectrophotometer and Qubit 2.0 (Thermo Fisher Scientific). DNA short-read libraries were prepared and sequenced using an Illumina HiSeq 2500 platform (Illumina) by Novogene. Long-read libraries were prepared using an in-house MinION sequencer by Oxford Nanopore Technologies, run for 24+ hours with base calling in the MinKNOW software on standard settings. Assembly was performed in CLC Genomics Workbench 20.0.4 (Supplementary Table 1), and all Illumina data was deposited at PRJEB42815 (ENA). Obtained MinION raw reads from one strain (Y72) were fully *de novo* assembled into three separate contigs representing the chromosome, the virulence plasmid, and one additional plasmid. The Nanopore sequence from Y72 was corrected using the trimmed Illumina reads for the same strain. The corrected Y72 assembly was used as a reference for mapping of trimmed Illumina reads for the remaining four strains (Supplementary Table 2).

### Preparing Reference Genomes

Illumina sequences were *de novo* assembled, and contigs were sorted by length. The five longest contigs (280--111 kbp) along with one additional 5.7-kb plasmid and a resistance cassette in Y72 were compared with genomic data available on NCBI using BLAST. As only a small set of WGS *Y. enterocolitica* genomes are available, the *Yersinia* EnteroBase V.1.1.2^1^ was used to identify available sequence data for *Y. enterocolitica* biotype 4 (Zhou et al., 2020). A total of 24 scaffold sets for different *Yersinia* 4/O:3 chromosomes, mainly based on a previous assortment (PRJEB2116 and PRJEB2117) (Reuter et al., 2014), were collected to increase phylogenetic resolution of the outbreak strains and comparative power of the metadata (Supplementary Table 3). Metadata included biotype, serotype, collection year, isolation country, isolation host, McNally ST, Achtman ST, wgMLST, and cgMLST + HierCC V1 (Supplementary Table 3). Based on high-score BLAST hits, an additional 17 different sequences of chromosomal and plasmid origin were collected for cassette comparisons (Supplementary Table 4) along with a set of three different alignment matches for the 5.7-kb plasmid.

### Functional Annotation

Molecular characterization for the outbreak strains and Y11 (six strains) were assessed with regard to pathogenicity potential, restriction--modification systems, and known virulence factors. Antimicrobial resistance genes were characterized in outbreak strains and the generated comparison platform (24 additional strains). Pathogenicity potential was assessed using PathogenFinder 1.1^2^ with the automatic model selection for assembled genomes (Cosentino et al., 2013). Antimicrobial resistance was assessed using ResFinder 4.1^3^ for acquired antimicrobial resistance genes among “other” species for assembled genomes (Zankari et al., 2017; Bortolaia et al., 2020). Restriction--modification systems were identified using Restriction-ModificationFinder 1.1^4^ for type I–IV restriction enzymes including putative genes (Roer et al., 2016). Virulence was assessed using the Virulence Factor Database VFDB^5^ for *Y. enterocolitica* (Chen et al., 2005, 2016; Yang et al., 2008; Liu et al., 2019).

### Phylogenetic Analysis

The 29 strains of *Y. enterocolitica* biotype 4 and one strain of biotype 2 (outgroup) were selected and analyzed on EnteroBase (Zhou et al., 2020). Enterobase’s cgMLST scheme was used to retrieve all SNPs from all strains. In total, 12,800 positions were polymorphic, of which about 3,600 were polymorphic in the ingroup. The alignment of all SNPs was used to infer a maximum-likelihood tree with IQ-TREE 2.1.2 (Minh et al., 2020). An extended range of substitution models was tested, and TVMe+ASC+R3, which had the lowest log likelihood according to Bayesian Information Criterion (BIC), was selected. This model assumes different rates for transitions and transversions, but with equal base frequencies (TVMe); it includes an ascertainment bias correction (ASC), which is appropriate in the case of SNP data (Lewis, 2001); it also includes a FreeRate model of rate heterogeneity across sites, with three categories (R3). A thousand ultrafast bootstraps (UFBoot) were drawn (Hoang et al., 2018).

All sequences matching the identified cassette were aligned to the known common ancestor *Shigella flexneri* R100 plasmid (Womble and Rownd, 1988), using the HOXD scoring-based Whole Genome Alignment plugin in CLC on standard settings (Chiaromonte et al., 2002). Only sequence regions overlapping with the *Yersinia* cassette were extracted (Extract Multiple Sequence Alignment tool) which corresponded to the R-det-Tn*2670* region of the R100 plasmid. As the 22 sequences shared different percentages of R-det coverage and the purpose of the comparison was to see the overall relationship among the transposon modules, a pairwise comparison (Create Average Nucleotide Identity Comparison) was generated with Alignment Percentage (AP) on standard settings. Based on the AP pairwise comparison, an AP similarity tree was constructed using Neighbor Joining (NJ), which joins clusters close to each other and far from other clusters, suitable for sequences with differential rates of evolution (Saitou and Nei, 1987). The AP NJ tree was rooted in the *Shigella flexneri* R100 plasmid. Both trees were later combined with collected metadata in CLC, and figure segments for Figures 2, 3 were combined in Serif’s Affinity Designer 1.9.1.979.

**FIGURE 1:**
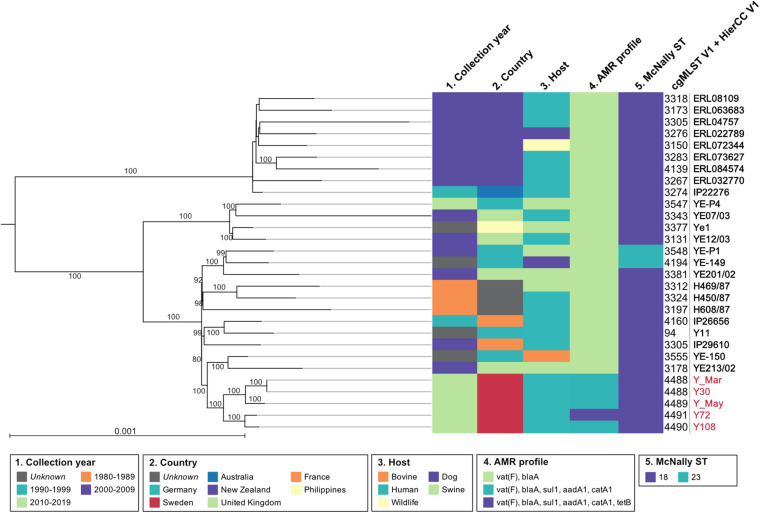
Phylogeny of the 29 *Y. enterocolitica* 4/O:3. The maximum-likelihood tree is based on all polymorphic positions identified by cgMLST in 30 genomes, including and rooted in an isolate from biovar 2 used as an outgroup, and not shown in the tree. Numbers over branches are the percentage of ultrafast bootstraps supporting that branch; support values <80 are not represented. The scale represents the average number of substitutions per site. Metadata for collection year, country, host, AMR profile, McNally ST, and cgMLST V1 + HierCC V1 were presented using CLC Genomics Workbench.

**FIGURE 2:**
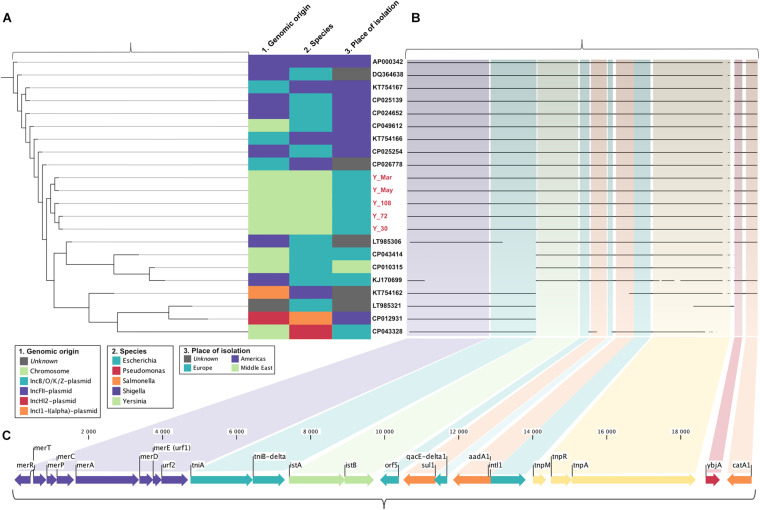
Schematic representation of the multidrug-resistant Tn*2670* transposon in clinical *Y. enterocolitica* and of similar mobile elements identified through BLAST. Segments were generated in CLC Genomics Workbench and combined in Affinity Designer. **(A)** Alignment Percentage Neighbor Joining relationship tree based on pairwise comparison of sequence alignments. **(B)** Sequence alignment of the Tn*2670* transposon indicating missing segments. **(C)** The Tn*2670* transposon depicted as situated in the chromosome of *Y. enterocolitica* Y72. Arrows indicate the orientation of the ORFs. Antibiotic resistance determinants are portrayed in orange and the mercury resistance operon in purple.

**FIGURE 3:**
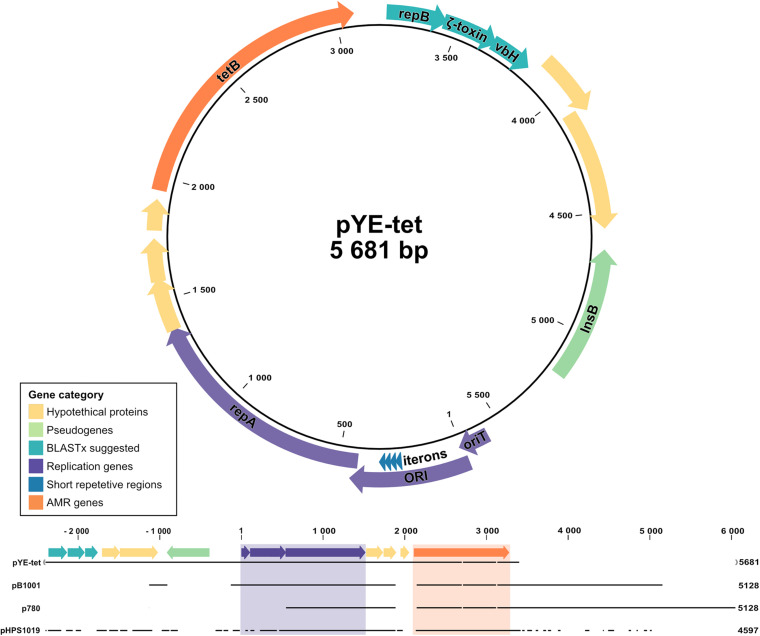
Schematic illustration and sequence alignment of pYE-tet plasmid in *Y. enterocolitica* strain Y72. Colors indicate gene category with replication-related elements presented in purple, antibiotic resistance gene *tet*B in orange, pseudogene *InsB* in green, xBLAST-suggested proteins in turquoise, and hypothetical proteins in yellow. Segments were generated in CLC Genomics Workbench and combined in Affinity Designer.

### Antimicrobial Susceptibility Testing

Minimum inhibitory concentration (MIC) tests were performed in biological duplicates with microdilutions in microtiter 96-well plates, where the antibiotic was added to the first well and diluted 1:2 per well in 10 consecutive wells. One column per plate was used as a positive control (no antibiotic), and one was used as a negative control (no bacteria). Bacterial concentrations of 0.5 McFarland standard units were added as 50 μl to each well. The starting concentrations were set to 2 × the clinical breakpoint (CB) for Enterobacterales (EUCAST). If no CB was available, E-tests were used as indicators for MIC and later adapted to broth microdilutions. All broth microdilutions were carried out in standardized EUCAST settings in Mueller Hinton media, incubated at 37°C for 16–20 h. Results were only accepted if controls were functional and the duplicates did not deviate more than one dilution.

### Growth Rate Measurements

Growth rate was used as an indicator for bacterial fitness and measured using the Bioscreen C (Oy Growth Curves Ab Ltd.) turbidity monitoring system. The bioscreen was performed in 300 μl TSB (1:1,000 dilutions from overnight culture) in biological triplicates with technical duplicates. Experiments were run at 26°C, with continuous shaking at medium amplitude and 195 rpm for 24 h. Measurement was done using a 600-nm brown filter every 4 min and shaking stop 5 s prior to measurement. Generation time was derived from the slope of the exponential growth phase (0.02 < OD_600_ < 0.12) where *R*^2^ ≥ 0.98 per sample and SD ≤ 0.001 (slope) across replicates. Data were analyzed and presented using GraphPad Prism 9.

## Results

### Comparative Genomics

A total of five time-representative isolates were obtained from PHAS (Y30, Y72, Y108) and Uppsala University Hospital (Y_Mar, Y_May) during the time of the two outbreaks. A well-established clinical strain of the same bioserotype was used as a control (Y11).

Five strains were assembled to complete genomes, molecularly typed, and assessed together with Y11 with regard to pathogenicity and virulence. The genomes from the outbreaks had an average estimated probability score for human pathogenicity of 0.875 (chromosome) and were found matching 95–99 different families. Clinical strains carried similar known and putative Restriction-Modification systems (*n* = 4) as the Y11 strain, with two Type II restriction enzymes and two methyltransferases. Virulence factors were shared across the collected strains and included genes for adherence (*psa*, *yap*), invasion (*ail, inv*), proteases (*pla*), and chromosomal secretion systems. There were eight open reading frames (ORFs) for the O-antigen and 42 ORFs for the cluster I flagella (Supplementary Table 5). The Swedish outbreak strains differentiated from the Y11 strain by the presence of a chromosomal Type II Secretion System protein (*yts1O*) and genes for antimicrobial resistance (Table 1 and Figure 1).

**TABLE 1:** Strain characterization.

	**Outbreak**	**Biovar/serotype**	**Pathogenicity score (families)**	**Resistance genes**	**Chromosomal secretion systems**
Y11	NA	4/O:3	0.89 (96)	*vat*(F), *blaA*	T3SS (21 ORFs)
Y30	First	4/O:3	0.875 (98)	*sul1*, *aadA1*, *catA1*, *vat*(F), *blaA*	T2SS (*yts1O*), T3SS (21 ORFs)
Y72	Unknown	4/O:3	0.875 (99)	*sul1*, *aadA1*, *catA1*, *vat*(F), *blaA*, *tetB*	T2SS (*yts1O*), T3SS (21 ORFs)
Y108	Second	4/O:3	0.872 (95)	*sul1*, *aadA1*, *catA1*, *vat*(F), *blaA*	T2SS (*yts1O*), T3SS (21 ORFs)
Y_Mar	First^*a*^	4/O:3	0.875 (98)	*sul1*, *aadA1*, *catA1*, *vat*(F), *blaA*	T2SS (*yts1O*), T3SS (21 ORFs)
Y_May	Unknown	4/O:3	0.875 (98)	*sul1*, *aadA1*, *catA1*, *vat*(F), *blaA*	T2SS (*yts1O*), T3SS (21 ORFs)

*^*a*^As identified by this study.*

Assembled genomes were phylogenetically analyzed based on maximum likelihood from all polymorphic positions identified by cgMLST in EnteroBase and rooted using an isolate from biovar 2/O:9 (IP26766, France, 2002) as an outgroup. The Swedish outbreak strains clustered together, having the closest shared node with a swine isolate from the UK (YE213/02, 2002) and a bovine isolate from Germany (YE-150, unknown collection year) (Figure 1). On a larger scale, the Swedish cluster grouped with European isolates from Germany, the UK, and France, including Y11. As inferred by McNally ST typing, the most common ST-type for 4/O:3 was ST18, but Hierarchical Clustering of cgMLST (HierCC) confirmed high similarity multilevel clustering for the Swedish outbreak strains. Results from ResFinder moreover demonstrated the global and time-independent presence of previously well-studied *Y. enterocolitica vat*(*F*) and *blaA* resistance genes, conferring resistance against streptogramin and β-lactam antibiotics (Pham et al., 1991; Stock et al., 1999; Seoane and García Lobo, 2000). The Swedish outbreak isolates also displayed a previously unreported multidrug resistance pattern (Figure 1).

### Resistance Cassette

We report the fully sequenced genome of Y72, showing the presence of a chromosomal multidrug resistance cassette harboring determinants against quaternary ammonium compounds (*qacE-delta1*), heavy metal mercury (*mer* operon), phenicols (*catA1*), streptomycin (*aadA1*), and folate pathway antagonists (*sul1*) (PRJEB42815). This cassette was shown to be a known variant of the Tn*2670* transposon, originally identified in the resistance segment (R-det) on a *S. flexneri* R100 plasmid in the 1950s (Nakaya et al., 1960). The average coverage of the transposon was approximately half of the chromosome in all strains but Y72 where it remained 100% (Supplementary Table 2). To investigate if carrying Tn*2670* might come at a fitness cost, growth rates were measured to estimate generation time, but no difference could be observed when grown in TSB compared to Y11 at 26°C (Supplementary Figure 1). Tn*2670* is a self-transmissible transposon flanked by direct repeats IS1*a* and IS1*b* with two overlapping open-reading frames (ORFs) that create a transposase after translational frameshifting (Hanni et al., 1982; Womble and Rownd, 1988; Escoubas et al., 1994; Partridge and Hall, 2004; Partridge, 2011). As demonstrated in Figure 2C, Tn*2670* carries the identified *catA1* (chloramphenicol acyltransferase) and *ybjA* (acyl-CoA acyltransferase) as well as the entire movable Tn*21* transposon (Sun et al., 2016). Tn*21* is an independent transposon lined with two indirect imperfect repeats, IR*_*tnp*_* (*tnp* genes for transposition) and IR*_*mer*_* (*mer* operon for mercury resistance) (De la Cruz and Grinsted, 1982; Liebert et al., 1999). Inside Tn*21* lies the class I integron In2, bordered by the indirect imperfect repeats IR*_*i*_* and IR*_*t*_*, which on its own is not mobile due to a truncated *tniB* and lack of additionally required *tni* genes (Stokes and Hall, 1989; Brown et al., 1996). In2 can in turn be subdivided into three distinct regions: the integron as a unit, the “*aadA1* cassette,” and an insertion sequence from a previous integron (IS*1326*). In2 can no longer transpose, but the integrase (*intl1*) can theoretically incorporate new genes at the *attl1* site, the location for adenyl transferase (*aadA1*), and dihydropteroate synthase (*sul1*) for streptomycin and sulfonamide resistance, respectively (Collis et al., 1993, 1998). The variant of Tn*2670* described here lacks IS*1353*, otherwise reported to locate inside IS*1326* (Grinsted and Brown, 1984; Brown et al., 1996) (Figure 2C).

The Tn*2670* transposon was compared with available sequences in NCBI using BLAST and analyzed using alignment percentage neighbor joining (Figures 2A,B). The transposon from Swedish strains clustered alone, but surrounded by *Escherichia* and *Shigella* carrying both plasmid-borne and chromosomally located sequence hits, mainly from plasmid types IncFII and IncB/O/K/Z. The cassette mainly matched sequences found in species of enteric bacteria, indicating interspecies spread and active dissemination of these determinants among *Enterobacteriaceae*.

### TetB Plasmid

Along with the multidrug resistance cassette, our analysis revealed an additional plasmid carrying tetracycline resistance in strain Y72 with a coverage approximately 16 × that of the chromosome (Supplementary Table 2). The plasmid, hereafter termed pYE-tet (Figure 3), has a size of 5,681 bp and sporadically shares an approx. 5-kb segment with smaller plasmids identified in the family of *Pasteurellaceae*. The smaller plasmids have specifically been reported in *Actinobacillus pleuropneumoniae* (plasmid p780, MH457196.1), *Haemophilus parasuis* (plasmid pHPS1019, HQ622101.1), and *Pasteurella multocida* (plasmid pB1001, EU252517.1). These three plasmids were aligned with pYE-tet in Figure 3. The segment carries a suggested ORI transfer site (*traJ-II*), an ORI with four 21-bp iterons (4 × TTATACGACTAGAAATTTCCTG), a replication protein (*repA*), a tetracycline resistance gene (*tet*B), and five hypothetical proteins (San Millan et al., 2009; Li et al., 2018). The hypothetical protein nucleotide sequences were run through BLASTx and gave hits on another replication protein (*repB*) (qq: 100%, id: 100%, AXF94983.1), a vbH antitoxin (qq: 100%, id: 100%, WP_119774164.1), and a gene with partial agreement to the *E. coli* zeta toxin (qq: 94%, id: 36%, EFC6518787.1). An approx. 700-bp segment was identified as a pseudogene for an IS*1ab InsB* transposase shared with several families within the order Enterobacterales, including *Morganella morganii* (CP026651.1), *Shigella dysenteriae* (CP026778.1), *Klebsiella pneumoniae* (CP047701.1), *S. enterica* (CP049986.1), *Proteus vulgaris* (CP047346.1), *Citrobacter freundii* (CP047247.1), and *Enterobacter hormaechei* (LC590026.1). The *tet*B sequence was shared between both enteric bacteria and *Pasteurellaceae*.

### Phenotypic Analysis of *Y. enterocolitica* Clinical Strains

Minimum inhibitory concentrations were assessed through broth microdilutions (Table 2). All outbreak strains were resistant to fusidic acid (>256 μg/ml), rifampicin (8 μg/ml), ampicillin (32 μg/ml), and erythromycin (64–128 μg/ml). Matching the characterized genotype was high-level resistance against chloramphenicol (>32 μg/ml), streptomycin (375–750 μg/ml), sulfamethoxazole (500 μg/ml), and tetracycline (32 μg/ml for Y72).

**TABLE 2:** Minimum inhibitory concentrations (μg/ml) against routinely used antibiotics.

	**FUS**	**RIF**	**AMP**	**CHL**	**STM**	**SMX**	**ERY**	**TET**	**CTX**	**CAZ**	**FOF**	**PIP**	**CIP**	**CST**	**GEN**
Y11	>256	8	4	<8	<24	<8	4	<4	<2	<8	<32	<16	<0.5	<8	<4
Y30	>256	8	32	>32	375	500	64	<4	<2	<8	<32	<16	<0.5	<8	<4
Y72	>256	8	32	>32	375	500	128	32	<2	<8	<32	<16	<0.5	<8	<4
Y108	>256	8	32	>32	375	500	64	<4	<2	<8	<32	<16	<0.5	<8	<4
Y_Mar	>256	8	32	>32	750	500	64	<4	<2	<8	<32	<16	<0.5	<8	<4
Y_May	>256	8	32	>32	375	500	64	<4	<2	<8	<32	<16	<0.5	<8	<4

*FUS: fusidic acid, RIF: rifampicin, AMP: ampicillin, CHL: chloramphenicol, STM: streptomycin, SMX: sulfamethoxazole, ERY: erythromycin, TET: tetracycline, CTX: cefotaxime, CAZ: ceftazidime, FOF: fosfomycin, PIP: piperacillin, CIP: ciprofloxacin, CST: colistin, GEN: gentamicin.*

## Discussion

In the spring of 2019, the Swedish Public Health Agency and Statens Serum Institut in Denmark independently identified an outbreak caused by *Y. enterocolitica* 4/O:3. Sequence comparison and epidemiological investigation confirmed this cross-border outbreak, which was associated with imported fresh spinach. Here we determined the genome sequences, predicted virulence and pathogenicity factors, and charted the antimicrobial resistance profile of five *Y. enterocolitica* clinical isolates appearing during the time of the Swedish outbreaks. In comparison, we included a well-characterized clinical strain (Y11) isolated in Germany.

Of the five isolates, all expressed the virulence plasmid of *Yersinia* (pYV) along with genes commonly associated with infection, including the *myf* operon, *yadA*, *ail*, and *invA* (Bancerz-Kisiel et al., 2018). A predicted pathogenicity score averaged at 87.5% for chromosomally encoded genes when compared to 95–99 available families. All strains carried a Type II restriction enzyme (M.YenYEP1ORF12551P/M.YenY11ORF26101P) and a Type II methyltransferase (YenY11ORF26101P) along with a putative Type II restriction enzyme (M.SmaB3R3ORF2440P) and putative methyltransferase (Yen002ORF2900P). Our strains from 2019, but not Y11, carried a prepilin-like peptidase (aspartic hydrolase), Yts1O, from the Type II Secretion System Yts1 (occasionally termed as Yst). The Yts1 Type II Secretion cluster has previously been linked to increased pathogenicity in *Y. enterocolitica*, but interestingly our outbreak strains only carried *yts1O*, without any of the remaining operon coding units for the Yts secreton. The Yts1 and Yts2 clusters have only recently been studied, and little is known about the function and molecular mechanisms related to the operons (Iwobi et al., 2003; Shutinoski et al., 2010; von Tils et al., 2012; Rusak et al., 2017). Prepilin peptidases share high sequence homology among bacteria and are known transmembrane proteins allocated to a wide set of functions including pilus biogenesis and Type II Secretion (Schreiber and Donnenberg, 2002; Dupuy et al., 2013). The exact role of Yts1O in the Yts1 cluster, and specifically here as a single enzyme, remains a pressing question for further investigation.

All five strains showed antimicrobial resistance to chloramphenicol (>32 μg/ml), streptomycin (375–750 μg/ml), and sulfamethoxazole (500 μg/ml), and strain Y72 additionally carried clinically relevant resistance against tetracycline (32 μg/ml). All outbreak strains, but not Y11, showed resistance against ampicillin (32 μg/ml) and erythromycin (64–128 μg/ml). While there is resistance against ampicillin and erythromycin which are two of the more commonly reported AMR phenotypes in 4/O:3, tetracycline resistance is only rarely reported (Fàbrega et al., 2015; Gkouletsos et al., 2019). Resistance against chloramphenicol, streptomycin, and sulfamethoxazole correlated well with the presence of *catA1*, *aadA1*, and *sul1* resistance genes situated on the chromosomally located Tn*2670* transposon (Figure 2). Moreover, resistance genes other than *vat*(*F*) and *blaA* appear rare incidents among 4/O:3 *Y. enterocolitica* (Figure 1; Pham et al., 1991; Stock et al., 1999; Seoane and García Lobo, 2000). This transposon, originally derived from the *S. flexneri* R100 plasmid (belonging to IncFII incompatibility group), has been shown transferable between plasmids *in vitro* by P7 phages (Nakaya et al., 1960; Hanni et al., 1982; Partridge and Hall, 2004). The internal transposon Tn*21* has been described widely spread among soil bacteria in both mercury-polluted and unpolluted sites and among clinically relevant Gram-negative bacteria, but to the best of our knowledge never before in *Yersinia* (Pearson et al., 1996; Liebert et al., 1999; Turner et al., 2003; Herrero et al., 2008; Partridge et al., 2018). Although both excision and loss rates are higher for composite transposons like Tn*2670* compared to isolated insertion sequences, it remains intriguing that the transposon coverage is about half that of the chromosome for all strains but one (Wagner, 2006; Sun et al., 2016). The fact that this known resistance cassette has never been reported in *Yersinia*, while found in all five independent isolates in this study, may indicate a new trend of antimicrobial resistance genes in European foodborne *Y. enterocolitica*.

The tetracycline resistance observed in strain Y72 was accompanied by a small *tet*B plasmid, pYE-tet, sharing genes with small plasmids from *Pasteurellaceae*. This pattern of genes on a small *tet*B plasmid was first reported in multidrug-resistant *P. multocida* (pB1001) from diseased pigs in Spain 2002–2005 (San Millan et al., 2009), later in *H. parasuis* (pHPS1019) from Chinese pigs in 2010 (Liu and He, 2010), and most recently in *A. pleuropneumoniae* (p780) from Brazilian pigs collected between 2006 and 2011 (Pereira et al., 2016; Li et al., 2018). Except for the more diverse zoonotic *P. multocida*, the previous carriers of such plasmids have all been bacteria restricted to swine. All three species colonize the upper respiratory tract of pigs where they share the same environmental niche as *Yersinia* (Oliveira and Pijoan, 2004; Reiner et al., 2010; Wilson and Ho, 2013). The *tet*B gene found on pYE-tet, pB1001, pHPS1019, and p780 has previously been speculated to originate from Gram-negative enteric bacteria, strengthened by previous horizontal gene transfer events of AMR genes from enterobacteria to *Pasteurellaceae* plasmids (Li et al., 2018; Michael et al., 2018). Both pB1001 and p780 have been described to lack standard mobilization genes (*mob*), and even though non-selectively and stably replicated in *Escherichia coli* following electroporation, no actual conjugation has been observed (San Millan et al., 2009; Li et al., 2018). Detailed work on the p780 plasmids revealed a 22-bp iteron Rep protein-binding site in ORI; although slightly different and one nucleotide shorter, this was also identified in pYE-tet (4 × TTATACGACTAGAAATTTCCTG). Preceding the ORI is, similar to p780, an ORI transfer site with a speculated secondary sRNA *traJ-II* structure with a *nic* cleavage site, suggesting specificity to TraJ-RP4 relaxomes (Figure 3). None of the plasmids encode their own relaxomes, and RP4 was previously illustrated insufficient for conjugation of the *Pasteurellaceae* plasmid in *E. coli*, leaving the mechanistic accounts behind plasmid transfer unexplained (Li et al., 2018).

Y30, isolated from the spinach-related outbreak, shared the same cgMLST as Y_Mar, which might well be related to the same outbreak. These two strains are in turn more similar to Y_May than to the two strains Y108 and Y72, supporting the previous notion that these in fact do not share the same spinach-related origin (Figure 1; National Veterinary Institute (SVA), 2019). Y72 carries the additional pYE-tet plasmid with a strong phylogenetic tie to species naturally occurring in the upper respiratory tract of swine, suggesting that this strain might be derived from an animal source instead. This proposition remains speculative, but further investigations on the stability (losing rate) and fitness cost of pYE-tet could provide valuable insight on time since parting with the HGT niche. The Swedish isolates may be of different origins, but all carry the Tn*2670* multidrug-resistant transposon, making it of even greater concern on food safety and public health. Although harboring an active large cassette (all strains) and the pYE-tet plasmid with a coverage approximately 16 × that of the chromosome (Y72), no growth rate differences could be observed as compared to Y11, which lacks all the mentioned elements. Our results suggest that these mobile elements do not come at a fitness cost at 26°C.

Neither the Tn*2670* transposon nor the small *tet*B resistance plasmid has been reported in foodborne *Yersinia* or in isolates derived from RTE products. The results presented here suggest horizontal gene transfer events in environment, agriculture, or animal husbandry, permitting *Y. enterocolitica* to be an additional foodborne carrier of multi-antibiotic and metal-resistance determinants. Prospective studies are needed to elucidate the mechanistic conjugative properties of pYE-tet, the stability and potential mobility of the Tn*2670* transposon, and the prevalence of these elements in clinical and food-related *Yersinia*. Our study highlights the concerns of food-chain contamination as potential reservoir for transmission and dissemination of AMR, raising concerns on food safety and public health.

## Data Availability Statement

The datasets presented in this study can be found in online repositories. The names of the repository/repositories and accession number(s) can be found in the article/Supplementary Material.

## Author Contributions

PK and HW: conceptualization and writing—original draft. CJ, ET, PK, and HW: data curation. PK, LG, and HW: formal analysis. HW: funding acquisition. PK, CJ, and HW: investigation. PK, RH, LG, and HW: methodology. HW: project administration. CJ, ET, LG, JJ, and HW: validation. ET, CJ, RH, LG, JJ, and HW: writing—review and editing. All authors contributed to the article and approved the submitted version.

## Conflict of Interest

The authors declare that the research was conducted in the absence of any commercial or financial relationships that could be construed as a potential conflict of interest.

## Footnotes

1 https://enterobase.warwick.ac.uk/species/index/yersinia

2 https://cge.cbs.dtu.dk/services/PathogenFinder/

3 https://cge.cbs.dtu.dk/services/ResFinder/

4 https://cge.cbs.dtu.dk/services/Restriction-ModificationFinder/

5 http://www.mgc.ac.cn/VFs/main.htm
