# Supplementary material for: Molecular Characterization of Multidrug-Resistant Yersinia enterocolitica From Foodborne Outbreaks in Sweden
Source: Front Microbiol. 2021 May 13;12:664665. doi: 10.3389/fmicb.2021.664665 (PMC8155512; doi:10.3389/fmicb.2021.664665)
Supplement: Supplementary file 1 [file Data_Sheet_1.docx]

Supplementary Material

# Supplementary Tables and Figures

## Supplementary Tables

**Supplementary table 1. Assembly metrics**

|  | N_50_ (Illumina) | N_50_ (Nanopore) | GC content | Mapped reads (%) | Chromosome length | pYV length |
| --- | --- | --- | --- | --- | --- | --- |
| Y30 | 70872 | NA | 47.2 | 98.17 | 4566927 | 71761 |
| Y72 | 66277 | 20 385 | 47.1 | 96.56* | 4567629 | 71773 |
| Y108 | 66729 | NA | 47.3 | 96.51 | 4566980 | 71774 |
| Y_Mar | 70922 | NA | 46.9 | 99.58 | 4567178 | 71761 |
| Y_May | 66664 | NA | 46.9 | 99.57 | 4565072 | 71768 |

*Illumina reads mapped to Nanopore reference

**Supplementary table 2. Illumina average coverage**

|  | Chromosome | pYV | Copy number | Cassette | Copy number | pYE-tet | Copy number |
| --- | --- | --- | --- | --- | --- | --- | --- |
| Y30 | 388.8 | 734.8 | 1.9 | 202.1 | 0.5 | NA | NA |
| Y72 | 321.5 | 634.1 | 2.0 | 397.3 | 1.2 | 5155.2 | 16.0 |
| Y108 | 329.3 | 793.6 | 2.4 | 199.6 | 0.6 | NA | NA |
| Y_Mar | 256.3 | 650.7 | 2.5 | 188.2 | 0.7 | NA | NA |
| Y_May | 279.6 | 523.7 | 1.9 | 155.5 | 0.6 | NA | NA |

## Supplementary table 3. Reference genomes

| Name | Accession | Country | Year | Host | Sample | McNally | Achtman | wgMLST | cgMLST V1 + HierCC V1 |
| --- | --- | --- | --- | --- | --- | --- | --- | --- | --- |
| IP29610 | ERR163908 | France | 2008 | Human | Stool | 18 | 135 | 4213 | 3305 |
| IP22276 | ERR163906 | Australia | 1991 | Human | Stool | 18 | 135 | 4198 | 3274 |
| IP26656 | ERR163907 | France | 1999 | Human | Stool | 18 | 135 | 5149 | 4160 |
| YE12_03 | HF933425 | UK | 2003 | Human | Stool | 18 | 135 | 4066 | 3131 |
| YE07_03 | ERR024570 | UK | 2003 | Human | Stool | 18 | 135 | 4281 | 3343 |
| YE213_02 | ERR024566 | UK | 2002 | Pig | NA | 18 | 135 | 4127 | 3178 |
| YE201_02 | ERR024584 | UK | 2002 | Pig | NA | 18 | 135 | 4296 | 3381 |
| H450_87 | ERR024888 | NA | 1987 | Human | NA | 18 | 135 | 4254 | 3324 |
| H469_87 | ERR024891 | NA | 1987 | Pig | NA | 18 | 135 | 4250 | 3312 |
| H608_87 | ERR024592 | NA | 1987 | Human | NA | 18 | 135 | 4109 | 3197 |
| ERL04757 | ERR024572 | New Zealand | 2004 | Human | Blood | 18 | 135 | 4222 | 3305 |
| ERL073627 | ERR024579 | New Zealand | 2007 | Human | Blood | 18 | 135 | 4196 | 3283 |
| ERL084574 | ERR024864 | New Zealand | 2008 | Human | Blood | 18 | 135 | 5139 | 4139 |
| ERL08109 | ERR024580 | New Zealand | 2008 | Human | Blood | 18 | 164 | 4238 | 3318 |
| ERL072344 | ERR024576 | New Zealand | 2007 | Wild animal | Blood | 18 | 135 | 4083 | 3150 |
| ERL063683 | ERR024867 | New Zealand | 2006 | Human | Blood | 18 | 185 | 4094 | 3173 |
| ERL022789 | ERR024575 | New Zealand | 2002 | Dog | NA | 18 | 135 | 4209 | 3276 |
| ERL032770 | ERR024863 | New Zealand | 2003 | Human | Blood | 18 | 135 | 4194 | 3267 |
| YE-P1 | GCF000401975 | Germany | 2003 | Pig | NA | 23 | 135 | 4487 | 3548 |
| YE-P4 | GCF000401955 | Germany | 2011 | Pig | NA | 18 | 135 | 4489 | 3547 |
| YE-149 | GCF000401935 | Germany | NA | Dog | NA | 23 | 135 | 5197 | 4194 |
| YE-150 | GCF000401995 | Germany | NA | Calf | NA | 18 | 135 | 4488 | 3555 |
| Ye1 | AGQO01000012 | Philippines | NA | Pig | NA | 18 | 135 | 4305 | 3377 |
| Y11 | GCF000253175 | Germany | NA | Human | Feces | 18 | 255 | 188 | 94 |
| Y30 | ERS5652595 | Sweden | 2019 | Human | Feces | 18 | 135 | 5583 | 4488 |
| Y72 | ERS5652596 | Sweden | 2019 | Human | Feces | 18 | 135 | 5587 | 4491 |
| Y108 | ERS5652597 | Sweden | 2019 | Human | Feces | 18 | 135 | 5586 | 4490 |
| Y_Mar | ERS5652598 | Sweden | 2019 | Human | Feces | 18 | 135 | 5585 | 4488 |
| Y_May | ERS5652599 | Sweden | 2019 | Human | Feces | 18 | 135 | 5584 | 4489 |

**Supplementary table 4. R-det/Tn2670 comparison strains**

| Name | Species | Location | Plasmid type | Year | Country | Host | Sample |
| --- | --- | --- | --- | --- | --- | --- | --- |
| AP000342 | Shigella flexneri | R100 plasmid | IncFII | 1958 | Japan | NA | NA |
| DQ364638 | Escherichia coli | NR1 plasmid | IncFII | 1980 | NA | NA | NA |
| CP025139 | Escherichia coli | pBH100-alpha plasmid | IncFII | 1974 | Brazil | Human | Urine |
| KT754167 | Shigella dysenteriae | p69-3818 plasmid | IncB/O/K/Z | 1969 | Guatemala | NA | NA |
| CP049612 | Escherichia coli O157:H7 | Chromosome | NA | NA | USA | Human | Feces |
| KT754166 | Shigella dysenteriae | p92-9000 plasmid | IncB/O/K/Z | 1991 | Panama | NA | NA |
| CP026778 | Shigella dysenteriae | 69-3818 plasmid | IncB/O/K/Z | NA | NA | NA | NA |
| LT985306 | Escherichia coli | RCS88 plasmid | IncFII | NA | NA | NA | NA |
| CP025254 | Escherichia coli | pBH100-alpha plasmid | IncFII | 1974 | Brazil | Human | Urine |
| CP024652 | Escherichia coli | pBH100-1 plasmid | IncFII | 1974 | Brazil | Human | Urine |
| ERS5652595 | Yersinia enterocolitica Y30 | Chromosome | NA | 2019 | Sweden | Human | Feces |
| ERS5652596 | Yersinia enterocolitica Y72 | Chromosome | NA | 2019 | Sweden | Human | Feces |
| ERS5652597 | Yersinia enterocolitica Y108 | Chromosome | NA | 2019 | Sweden | Human | Feces |
| ERS5652598 | Yersinia enterocolitica Y_Mar | Chromosome | NA | 2019 | Sweden | Human | Feces |
| ERS5652599 | Yersinia enterocolitica Y_May | Chromosome | NA | 2019 | Sweden | Human | Feces |
| KJ170699 | Escherichia coli | K12 plasmid | IncFII | 2004 | Netherlands | Chicken | Broilery |
| CP010315 | Escherichia coli | Chromosome | NA | 1990 | Israel | Turkey | Blood |
| LT985321 | Escherichia coli | RCS85 plasmid | NA | NA | NA | NA | NA |
| CP012931 | Salmonella enterica Heidelberg | pN13-01290_23 plasmid | IncHI2 | 2012 | Canada | Turkey | meat |
| CP043414 | Escherichia coli | Chromosome | NA | 2015 | France | Bovine | NA |
| KT754162 | Shigella dysenteriae | BU53M1 plasmid | IncI1-I(alpha) | NA | NA | NA | NA |
| CP043328 | Pseudomonas aeruginosa | Chromosome | NA | 2001 | Sweden | Human | Urine |

**Supplementary table 5. Virulence factors**

|  | Adherence | Invasion | O-antigen | Proteases | Flagella | Restriction-Modification Systems |  | Plasmid of Yersinia virulence |
| --- | --- | --- | --- | --- | --- | --- | --- | --- |
| Y11 | **Myf/pH6**, **YapE** | **Ail**, **Invasin** | 8 ORFs | **Pla** | 42 ORFs (cluster I) | **Type II** M.YenYEP1ORF12551P, YenY11ORF26101P, Yen002ORF2900P*, M.SmaB3R3ORF2440P* |  | **YadA**, **Ysc-Yop System**, (41 genes)** |
| Y30 | **Myf/pH6**, **YapE** | **Ail**, **Invasin** | 8 ORFs | **Pla** | 42 ORFs (cluster I) | **Type II** M.YenYEP1ORF12551P, YenY11ORF26101P, Yen002ORF2900P*, M.SmaB3R3ORF2440P* |  | **YadA**, **Ysc-Yop System**, (41 genes)** |
| Y72 | **Myf/pH6**, **YapE** | **Ail**, **Invasin** | 8 ORFs | **Pla** | 42 ORFs (cluster I) | **Type II** M.YenYEP1ORF12551P, YenY11ORF26101P, Yen002ORF2900P*, M.SmaB3R3ORF2440P* |  | **YadA**, **Ysc-Yop System**, (41 genes)** |
| Y108 | **Myf/pH6**, **YapE** | **Ail**, **Invasin** | 8 ORFs | **Pla** | 42 ORFs (cluster I) | **Type II** M.YenYEP1ORF12551P, YenY11ORF26101P, Yen002ORF2900P*, M.SmaB3R3ORF2440P* |  | **YadA**, **Ysc-Yop System**, (41 genes)** |
| Y_Mar | **Myf/pH6**, **YapE** | **Ail**, **Invasin** | 8 ORFs | **Pla** | 42 ORFs (cluster I) | **Type II** M.YenYEP1ORF12551P, YenY11ORF26101P, Yen002ORF2900P*, M.SmaB3R3ORF2440P* |  | **YadA**, **Ysc-Yop System**, (41 genes)** |
| Y_May | **Myf/pH6**, **YapE** | **Ail**, **Invasin** | 8 ORFs | **Pla** | 42 ORFs (cluster I) | **Type II** (*M.YenYEP1ORF12551P, YenY11ORF26101P, Yen002ORF2900P*, M.SmaB3R3ORF2440P**) |  | **YadA**, **Ysc-Yop System**, (41 genes)** |

*putative

**no yscA

## Supplementary Figures

Growth rates were used as an indicator for fitness and measured with a bioscreen in 26°C, generation times (GT) were derived. All outbreak strains shared a similar GT at 26°C with no significant difference.


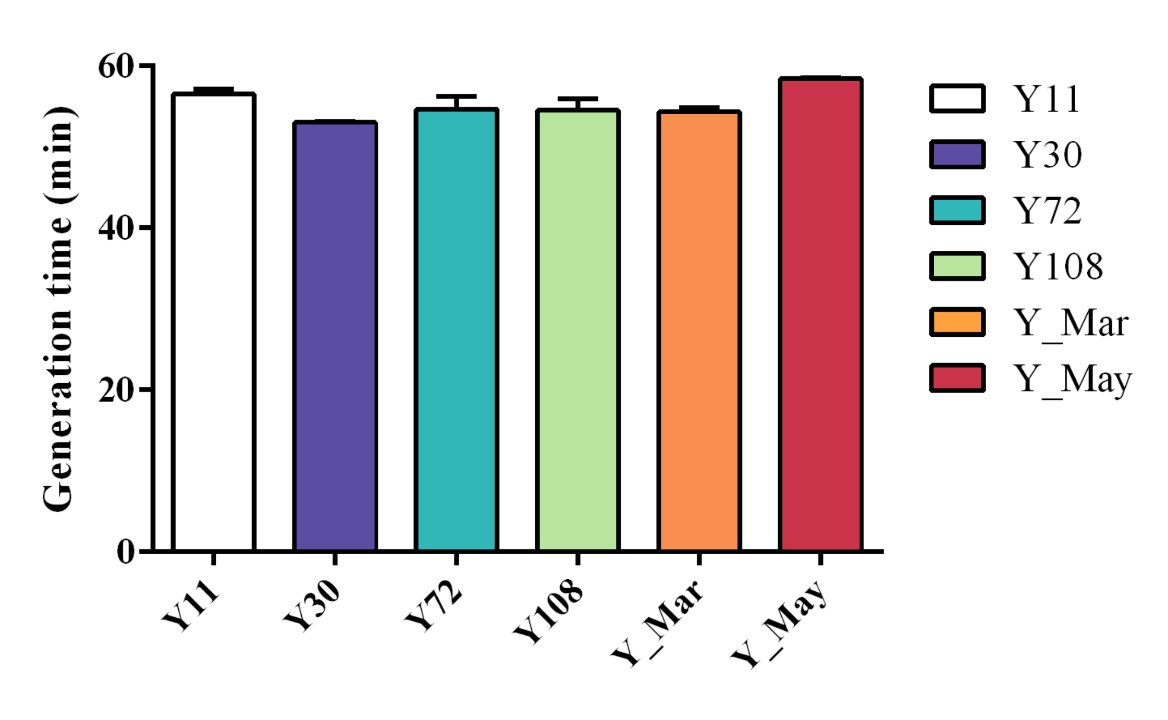


**Supplementary figure 1. Generation time of outbreak strains.**

Fitness as indicated by growth rate in calculated generation time (time for binary fission to occur). Experiment was run over 24h but generation time was derived from the exponential growth phase (OD >0.02, <0.12). Significance measured between outbreak strains and Y11 (t-test), error bars represent standard error of the mean (SEM).
